# Reliability of Measuring the Cervical Sagittal Translation Mobility with a Simple Method in a Clinical Setting

**DOI:** 10.1155/2012/629104

**Published:** 2012-11-04

**Authors:** Yvonne Severinsson, Lena Elisson, Olle Bunketorp

**Affiliations:** ^1^Department of Stomatognathic Physiology, Institute of Odontology, The Sahlgrenska Academy, University of Gothenburg, Gothenburg, Sweden; ^2^Department of Orthopaedics, The Institute of Clinical Sciences, The Sahlgrenska Academy, University of Gothenburg, Gothenburg, Sweden

## Abstract

*Introduction*. The cervical sagittal translation mobility is related to neck pain. A practical method for measuring the specific cervical mobility is needed. The aim was to describe a simple method for measuring the cervical sagittal translation mobility and to evaluate its reliability in a clinical setting. *Method*. The head protraction and retraction ranges of thirty healthy seated subjects were measured from a dorsal reference plane by two physiotherapists utilizing a tape measure. A standard inclinometer/goniometer was used to minimize angular movements of the head during the translational movements. The measurements were made twice for each subject with a two-hours interval between each measurement. The inter-rater and intra-rater agreements were evaluated with intraclass correlation coefficients (ICCs) and with the distribution of the difference of the measurements. The systematic differences were analysed with the Wilcoxon signed rank test. *Results*. The intra-rater agreement was good. The inter-rater agreement was moderate in the first measurement and good in the second. A systematic difference was noted between raters in the first measurement but not in the second, possibly indicating a learning effect. *Discussion*. The method used in the study is simple and reliable and can be recommended for clinical use.

## 1. Introduction

The biomechanics of the cervical spine is complex. Symptoms arising from the cervical spine vary, and researchers suggest that neck pain should be subdivided into upper cervical spinal pain and lower cervical spinal pain, above or below an imaginary transverse plane through C4 [1]. The cervical sagittal translation movements consist of protraction and retraction; protraction causing a flexion of the lower cervical spine and an extension of the upper cervical spine, and retraction the opposite [2, 3]. It has been suggested that these cervical movements are important in the rehabilitation of the neck [4–7]. Head posture is related to the natural configuration of the cervical spine [8]. Forward head posture can be defined when the center of gravity of the head is displaced ventral to the gravity line through the body of C7.

Neck pain is often associated with a restricted range of motion in the cervical spine [9–11], and several authors have studied the significance of head posture in subjects with neck pain [12–15]. Static forward head posture, such as having the head in a protracted position for long periods of time, causes posterior neck pain to develop in healthy subjects, according to Harms-Ringdahl and Ekholm [16]. There is also evidence that neck patients have significantly less range of the cervical sagittal translation mobility than normal subjects [13]. Cervical sagittal translation mobility has been studied in WAD (Whiplash Associated Disorders) patients and appears to be especially related to neck distortion in rear-end car impacts [17].

Clearly the mobility of the cervical spine is an important parameter in the physiotherapist's clinical work. An objective assessment of the active cervical range of motion (ACROM) requires a good measurement accuracy. Different measurement instruments have been tested for validity and reliability for ACROM, including flexion, extension, rotation, and side bending [18–25], and an inclinometer/goniometer is considered to be practical to use [22]. A practical method for measuring head posture [26, 27] and the total head excursion has also been presented [12, 13]. The range of cervical sagittal translation mobility has been assessed in radiographic studies [28–31]. However, to our knowledge, a simple and reliable method to accurately make such measurements in a clinical setting has been neither described nor tested. The aim of this study was to describe a simple method for the measurement of the cervical sagittal translation mobility and to evaluate its reliability in a clinical setting. 

## 2. Method

The study was conducted by two physiotherapists (A and B); both specialized in issues of the cervical spine, well trained for this purpose, and with extensive experience in the use of the measurement device. 

### 2.1. Subjects and Data Collection

Thirty healthy subjects, 21 women (mean age 44, range 26–64) and nine men (mean age 33, range 25–54), were included. Eleven women and three men, all physiotherapists, were recruited from the Department of Physiotherapy at Sahlgrenska University Hospital in Gothenburg. Six male dental students were recruited from the Institute of Odontology at The Sahlgrenska Academy in Gothenburg. Ten female dental nurses were recruited from a specialist clinic at the Public Dental Service in Gothenburg. All subjects were verbally informed of the purpose of the study and invited to participate. The only exclusion criterion was ongoing neck problems. The data collection was carried out during February and March 2009.

### 2.2. Measurement Equipment

A Myrin's inclinometer/ goniometer (Art. Nr. 711432, Bålsta, Sweden) was used. The instrument has an inclination needle, affected by gravity, that is used to measure side bending, flexion, and extension. It also has a compass needle that is used to measure horizontal rotation. The instrument was attached to the head by means of a Velcro strip. A metal tape measure was used to measure, in millimeters, the sagittal translation mobility during head protraction and retraction.

### 2.3. Procedure

The subjects were measured in random order, using identical equipment, arranged in the same way. Independently of each other, the raters A and B measured one subject each in the same room and on the same occasion, and the two subjects changed places immediately after the measurements. Each rater filled in their own protocol after testing each subject. All protocols were separated from each other and not checked between the measurements. Each subject was examined four times. The first two measurements of each subject were made before noon (12.00 P.M.) by A and B and were made within 30 minutes of each other. After two hours elapsed, the measurements were then repeated using the identical method and instrument as had been used for the first measurement.

The translation mobility of each subject was measured with the subject sitting on a stool close to a wall while maintaining good upright balanced posture, with both feet on the floor, with normal lumbar lordosis, hands on thighs, and with 90 degrees in the hip and knee joints. The subject was requested to assume a neutral head position, with the purpose of positioning the head's center of mass in a vertical plane through the atlantooccipital joints with the nose pointing forward in line with the sternum and bellybutton. A wedge-formed pad was fixed between the upper thoracic spine and the wall in an attempt to minimize the thoracic spine motions. The pad was placed with its upper edge at the spinous processes of C7-TH1. The inclinometer was placed above the right ear with its needle pointing vertically towards the centre of the external ear channel. The inclinometer was calibrated and held at zero in order to avoid head flexion/extension during these movements. 

**Figure 1:**
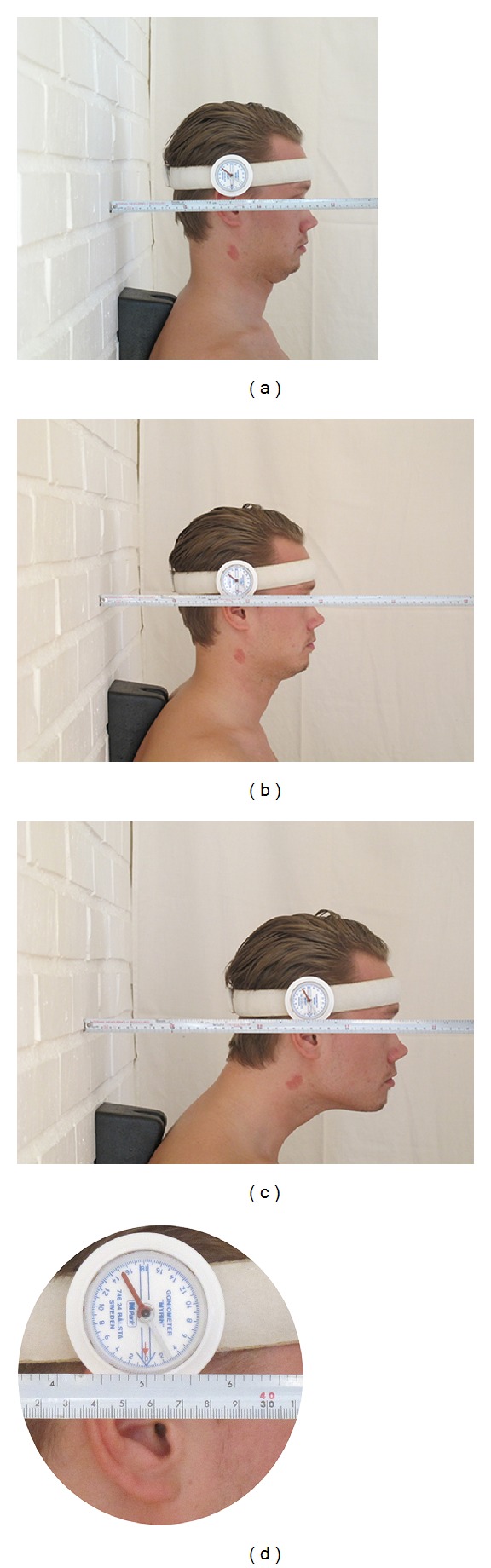
Measuring in the retracted, neutral, and protracted position of the head with the inclinometer and metal tape measure.

The distance between the wall and the vertical line of the inclinometer needle was measured with the metal tape measure with the subject's head in the neutral position. The end of the tape measure was placed so that it extended from the wall at a 90 degree angle and laid horizontally close to the needle (Figure 1). After measuring the neutral position, the subject was asked to protract the head maximally while the rater checked the inclinometer. In order to achieve a pure translation, divergences from the vertical line were corrected, in case a head flexion or extension occurred. If so, the needle indicated this movement and the rater guided the subject into the right position. At the maximum protraction, the distance between the needle and the wall was measured again (Figure 1). The subject then moved the head backwards to the neutral position. A similar procedure was used for retraction (Figure 1). The movements were performed once, and the values of the measurements were added to the protocol in centimeters carried to one decimal point. The difference between the maximum protraction and the maximum retraction ranges—the sagittal mobility (SM)—was calculated for each subject and then analyzed statistically. 

### 2.4. Ethical Considerations

Approval from the ethical committee was not applied for the study.

### 2.5. Calculations and Statistical Analyses

All data were compiled and analyzed with the standard version of SPSS 17.0 (Statistics Package for the Social Sciences, Chicago, IL, USA), except for the within-rater standard deviation (WRSD), which was calculated by use of the following formula [32]:

(1) $$\begin{array}{c} \text{WRSD}=\surd \left[\frac{{\text{SMdiff}}_{n}^{2}}{2*N}\right], \end{array}$$

where SMdiff_*n*_ = difference for the sagittal mobility between the first and second measurement for case *n*; *n* = 1, 2, …, *N*; *N* = 30.

Systematic differences between the two measurements of the sagittal mobility made by each rater and between the measurements made by the two raters were both analyzed with Wilcoxon Signed Rank Test for related samples. All significance tests were two-tailed and conducted at the 5% significance level.

The intra-rater and the inter-rater reliability were both described with the intraclass correlation coefficient (ICC), using the SPSS alfa two-way mixed model and consistency type. ICC values ≥0.81 were considered very good, 0.61–0.80 good, 0.41–0.60 moderate, and ≤0.40 fair or poor [33].

The 95% limits of the inter-rater agreements were calculated as the mean value of the differences between the raters ± 1.96 times the standard deviation of these differences. Bland-Altman plots and scatter plots of the differences between the measurements versus the means are given both for the inter-rater and the intra-rater analysis.

## 3. Results

The measurement data for protraction, neutral position, and retraction are presented in Table 1.

The statistical data for estimation of the intra-rater and the inter-rater agreement are presented in Table 2 and Table 3, respectively.

**Figure 2:**
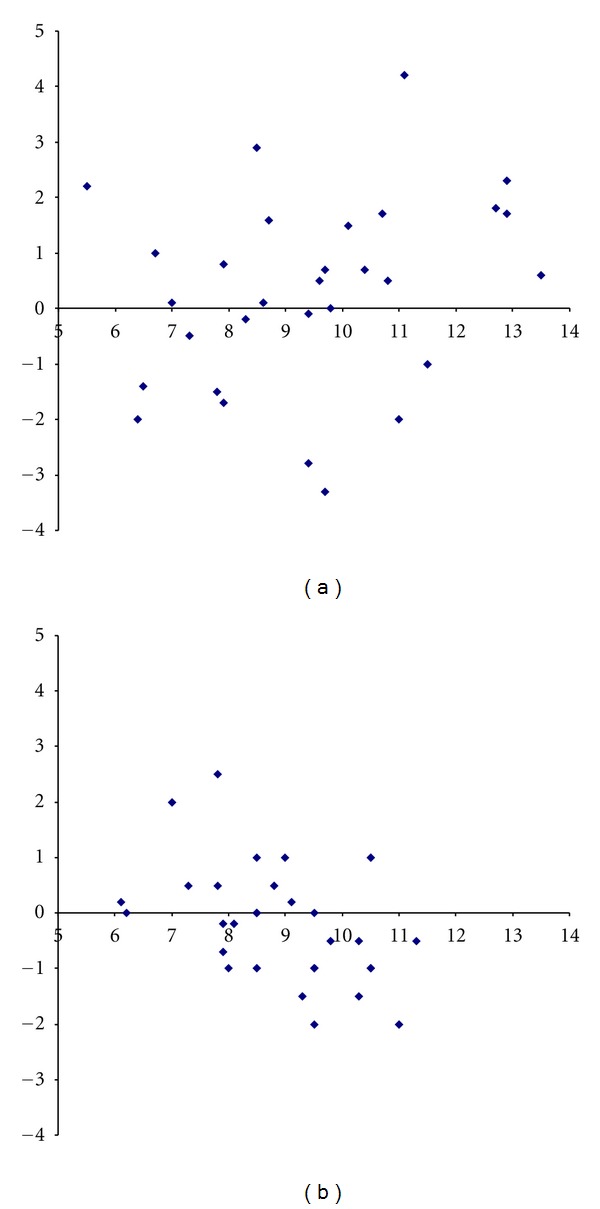
(a) Bland-Altman plot showing differences for the sagittal mobility (cm) at the two measurements (*Y*-axis) made by rater A versus the mean values of these measurements (*X*-axis). Every dot represents one subject. (b) Bland-Altman plot showing differences for the sagittal mobility (cm) at the two measurements (*Y*-axis) made by rater B versus the mean values of these measurements (*X*-axis). Every dot represents one subject.

The mean value of the sagittal mobility (SM; protraction minus retraction) for all measurements was 9.1 cm (range 4.4–14.0; SD = 1.9). The differences for SM measured by A on the two occasions, versus the mean values of SM, are shown in Figure 2(a) (intra-rater comparison). The differences were not correlated to the mean values for A (Pearson correlation coefficient = 0.26; *P* = 0.16).  Figure 2(b) shows the corresponding data for B, and in this case such a correlation was found (Pearson correlation coefficient = −0,48; *P* = 0.007).

**Figure 3:**
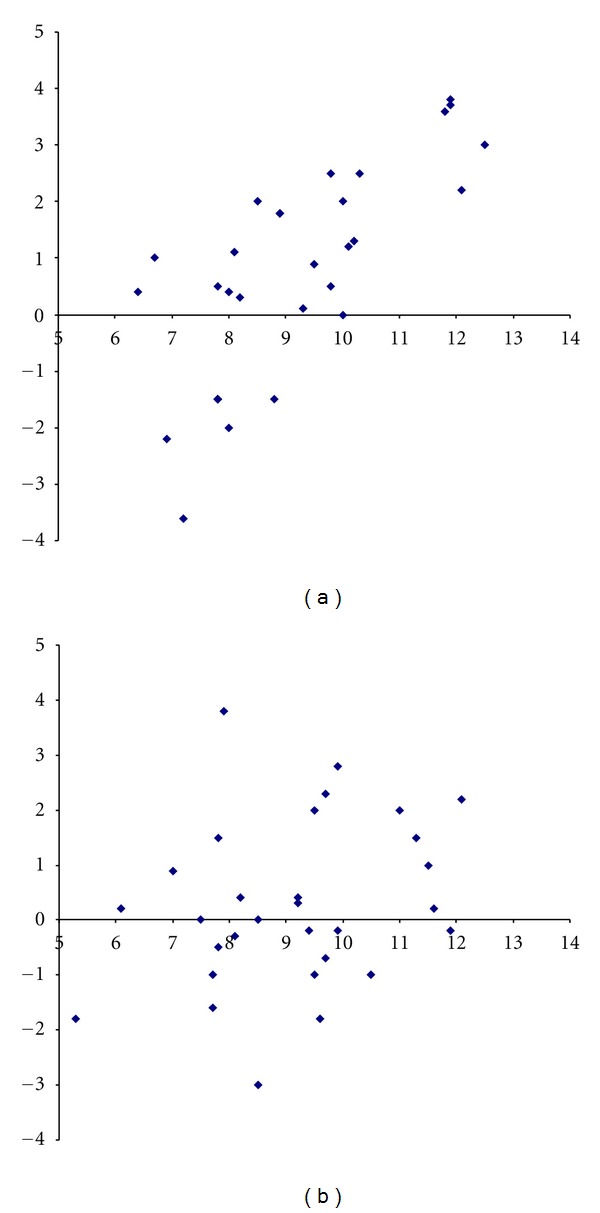
(a) Bland-Altman plot showing differences for the sagittal mobility (cm) at the first measurements (*Y*-axis) made by rater A and rater B versus the mean values of these differences (*X*-axis). Every dot represents one subject. (b) Bland-Altman plot showing differences for the sagittal mobility (cm) at the second measurements (*Y*-axis) made by rater A and rater B versus the mean values of these differences (*X*-axis). Every dot represents one subject.

The differences for SM measured by A and B on the first occasion versus the mean values are shown in Figure 3(a) (inter-rater comparison). These differences were significantly correlated to the mean values (Pearson correlation coefficient = 0.74; *P* < 0.001). The corresponding values for the second measurement are shown in Figure 3(b). No correlation was found between the differences and the mean values for the second measurements (Pearson correlation coefficient = 0.28; *P* = 0.14).

## 4. Discussion

This study describes a simple method for measuring the cervical sagittal translation mobility in a clinical setting, using an existing measuring device for cervical angular movements, combined with a metal tape measure. The instrument used for cervical angular movements in the study is well known and has been used since many years in Sweden. Similar instruments may exist on the market. We cannot see why these would give less accuracy using the same method, but this should be confirmed. 

The intra-rater agreement was good. The systematic difference was not statistically significant for either of the raters, and the intraclass correlation coefficient (ICC) was 0.7 for both of them (Table 2). The differences for A showed a somewhat greater variation (SD 1.72, range −3.3 : 4.2 cm) than for B (SD 1.07, range −2.0 : 2.5 cm). However, the differences for A were not correlated to the mean values (Figure 2(a)). Such a correlation was found for B (Figure 2(b)). The within-rater standard deviation was 1.2 cm for A and 0.8 cm for B (Table 2). The mean of these values (1.0 cm) is eleven percent of the mean value (9.1 cm) of the sagittal mobility. For A, the differences were less or equal to 1.5 cm in seventeen of the thirty cases (57%) and less or equal to 1 cm in fourteen cases (47%). For B, the differences between the measurements were less or equal to 1.5 cm in twenty-six of the thirty cases (87%) and less or equal to 1 cm in twenty-four cases (80%).

The inter-rater agreement was moderate and showed a systematic difference at the first examination (ICC = 0.53; *P* = 0.036; Table 3; Figure 3(a)). However, the differences between the raters varied widely (SD: 1.88 cm; 95% limits of agreement: −2.91; 4.47), and A recorded greater differences than B in most cases, especially at greater mobility (Figure 3(a)). The differences were less or equal to 1.5 cm in sixteen of the thirty cases (53%). The inter-rater agreement was good and without systematic difference at the second measurement (ICC = 0.67; *P* = 0.412; Table 3; Figure 3(b)). The SD for the difference was 1.5 cm (95% limits of agreement −2.7; 3.3). The differences were less or equal to 1.5 cm in twenty of the thirty cases (67%). A better agreement during the second measurements compared with the first ones may be an effect of what was learned by the raters during the first series of measurements.

Various circumstances may affect the reliability of the methodology employed in this study. Variations in the measurements taken by the raters, both inter-rater and intra-rater reliability, are due to factors relating to the subjects individually, the measurement instruments and the measurement process. The subject may differ in range of motion for various reasons. Some subjects may have had more neck or back stiffness in the morning because of pathological changes or muscle tension. Some subjects complained about neck pain after the first measurements, which could inhibit movements during the second measurements. Other factors are the motivation of the subjects, their ability to understand the instructions, and the raters' behaviour, that is, to inspire and instruct the subjects, or the subject's ability to make and repeat the movement in the same way [19, 21]. It is also important to standardize the position of the instrument on the head, to check the vertical pointer in order to adjust the head inclination during the horizontal movements when necessary, and to the place wedge correctly at C7-Th1 in order to avoid thoracic movements. Variation also depends on the sitting position, how the head is moved during the translation as well as during measurement of the distance between the wall and neutral position, which may vary each time a subject changes positions. A critical point was to keep the tape measure horizontal during the measurements. The use of a metal tape measure made this easier, and the authors do not think that small deviations from the horizontal position will influence the measurements so much.

Penning [2] demonstrated ten degrees greater extension during protraction in the upper cervical segments than in the other segments compared with normal full-length extension. He has also shown that the flexion ability in the upper cervical was ten degrees greater during retraction than during normal full-length flexion. Head translation in the sagittal plane also produces displacements in cervical-thoracic motion segments. Persson et al. [34] measured the total head excursion and found that approximately ten percent of the total head excursion originated from the thoracic regions in the sitting position. The authors tried to control for this bias by stabilizing the upper thoracic segments using a wedge for the subject to lean against. Thoracic movements may still have contributed to the measure differences between the raters in our study. This technical error was not observed by Hanten et al. [12, 13], who measured the total head excursion using a simple measurement method in normal and patient comparisons.

The reliability of nonradiological measurements of the cervical translation mobility has not been investigated sufficiently, and to our knowledge there is no reference for normative values, which have been estimated with a simple instrument in a clinical setting. The method presented in this study has been used for WAD patients [17]. Hanten et al. [12, 13] measured the total head excursion in the sagittal plane, and they concluded that the mean distance from the fully retracted to the fully protracted position was 7 cm for subjects with neck pain and 10.9 cm for subjects without neck pain; however, they did not test the reliability of their methodology. Hanten et al. [12, 13] also found that women had different head posture than men. In our study we investigated only healthy subjects without regard to age or gender and found that the mean distance from the fully retracted to the fully protracted position was 9.1 cm. Age and asymptomatic degenerative changes affect the cervical range of motion [20, 22]. The total cervical sagittal translation mobility has been measured on sagittal flexion radiographs and the authors found that women differed significantly from men [28, 30, 31]. Also a nonradiological study has shown that women have greater cervical translation mobility in the sagittal plane than men [34]. 

The authors consider that the cervical sagittal translation mobility is important when treating neck patients [4–7, 11–17, 27, 34]. A static protracted head posture for long periods is stressful to the anatomical structures of the cervical spine and causes pain and other symptoms, which may be related to the neck [16]. Clinical studies have shown that impaired neck rotation, extension, and neck retraction predict high disability [5, 6]. To unload the cervical spine from a static protracted position (forward head posture) and in that way improve other cervical movements and decrease neck pain, retraction exercise is a common treatment method used by physiotherapists [4–6]. Professionals dealing with these issues have been seeking a reliable instrument for measuring all cervical motions [35]. The authors can recommend the method used in the present study for the assessment of the cervical sagittal translation mobility in a clinical setting. However, there are limitations of the study. The number of subjects was quite small, and so was the number of measurements. Measurements were performed at different times and at different places. Further, recruiting subjects for the study was prolonged due to the scheduling conflicts of both the raters and those being recruited. Moreover, the short period of time available to complete the study possibly diminished the overall reliability. The reliability might be improved by employing a better standardized procedure and by ensuring that the same instructions were given to the study subjects. The reliability for this measurement method should also be confirmed with a greater number of subjects and raters.

## 5. Conclusions 

The method used in the study is simple and can be recommended for clinical use. The intra-rater agreement was good. The inter-rater agreement was moderate at the first measurement and good at the second one, indicating a learning effect. Further investigations with similar devices, used in clinical settings, are recommended in order to confirm the results.

## Figures and Tables

**Table 1:** Horizontal distance (cm) between the reference plane (the wall) and the measuring point (the Myrin inclinometer needle) during maximum active protraction, neutral position, and maximum active retraction for the two raters and the two measurements.

Case	Sex	Age	Rater A						Rater B					
			Measurement 1			Measurement 2			Measurement 1			Measurement 2		
			Protraction	Neutral	Retraction	Protraction	Neutral	Retraction	Protraction	Neutral	Retraction	Protraction	Neutral	Retraction
1	f	26	25.3	17.5	16.0	25.0	18.0	15.6	24.5	18.0	15.3	24.5	18.5	15.5
2	f	24	22.5	16.5	14.5	25.3	18.0	14.0	24.5	17.7	15.0	23.0	17.5	14.5
3	f	50	23.8	18.5	14.0	23.3	18.2	13.5	23.0	18.6	15.0	22.0	18.0	16.0
4	f	37	23.2	17.0	11.7	22.8	18.0	13.0	23.0	17.0	14.0	22.0	16.0	12.0
5	f	25	21.7	17.0	11.9	21.2	16.3	11.9	22.0	17.0	14.0	21.5	16.5	12.5
6	f	53	26.8	18.8	16.0	25.5	18.0	16.2	26.0	18.5	16.5	24.5	19.0	14.5
7	f	61	23.6	18.2	15.3	21.5	17.6	14.0	23.5	18.3	15.5	24.5	18.5	17.0
8	f	44	19.0	15.0	12.0	21.5	16.5	12.8	20.5	16.0	12.0	22.5	17.0	12.0
9	f	39	21.8	16.5	14.8	22.0	17.0	14.5	21.5	15.0	12.5	22.0	17.0	14.0
10	f	34	24.5	17.0	13.5	24.5	18.0	14.0	23.0	16.5	14.0	24.0	18.0	15.5
11	f	56	23.5	17.0	13.5	22.8	17.4	13.5	23.0	17.0	13.5	24.0	18.0	14.5
12	m	39	19.5	15.3	10.9	21.5	17.2	13.0	20.5	17.0	13.0	21.5	17.0	14.5
13	m	27	26.0	16.0	12.2	26.0	16.0	12.8	24.0	16.8	14.0	25.0	17.5	14.0
14	m	32	24.5	15.7	13.5	25.0	16.0	13.0	24.0	17.0	15.5	25.0	17.5	15.0
15	m	25	33.2	27.3	26.2	22.0	17.0	13.5	23.0	16.5	14.5	21.0	16.0	12.5
16	m	29	25.8	16.8	12.2	25.2	18.5	13.4	24.0	16.3	14.0	24.0	17.0	12.0
17	m	31	27.5	17.3	13.8	26.0	17.3	14.0	26.5	19.2	16.5	27.0	20.0	16.5
18	m	25	28.5	18.5	14.5	25.7	17.5	14.0	27.0	18.0	16.0	26.0	18.0	14.5
19	m	33	24.5	16.5	11.3	21.5	16.5	12.5	23.5	17.5	12.5	23.0	17.0	13.0
20	m	25	25.5	19.5	15.5	29.0	20.5	17.0	28.0	19.6	18.0	27.0	20.0	16.0
21	f	46	22.5	16.5	14.5	24.3	17.0	13.5	22.5	17.2	15.0	23.5	18.0	15.0
22	f	63	20.0	16.0	13.0	20.6	16.0	13.7	23.5	17.2	15.0	22.5	17.5	14.0
23	f	45	18.2	15.5	12.8	21.7	16.6	14.3	22.0	15.5	13.0	20.5	16.0	14.0
24	f	56	19.8	15.3	13.2	18.2	15.5	13.8	21.0	16.5	14.8	20.0	15.5	13.8
25	f	39	22.4	17.5	14.2	20.0	15.3	11.6	22.3	18.5	14.5	21.0	16.0	13.0
26	f	40	22.4	17.2	12.5	21.0	15.2	14.0	21.5	16.2	12.5	23.0	16.5	13.0
27	f	56	21.0	18.0	15.2	19.5	16.0	12.3	23.0	18.2	15.0	22.2	18.5	14.0
28	f	41	22.3	15.8	11.6	23.2	17.2	13.2	22.5	16.5	13.0	24.0	18.0	13.0
29	f	46	19.7	14.5	10.2	20.2	15.9	12.3	19.5	16.3	12.0	20.5	16.2	12.3
30	f	45	20.7	16.5	13.5	22.7	19.0	16.5	23.2	19.0	17.0	24.0	20.5	18.0

**Table 2:** Assessment of the intra-rater agreement for the cervical translation mobility in the sagittal plane for rater A and B. The mean values for the differences between the two measurements made by A and the two measurements made by B are presented together with its SD, medians, and ranges, as well as the *P* values for the systematic difference, the within-rater standard deviation, and the intraclass correlation coefficient (ICC).

Rater	*N *	Difference between the first and second measurements on each subject made by each of the raters (cm)					Systematic difference *P* value	Within-rater standard deviation (cm)	ICC
		Mean	SD	Median	Range	
					Min	Max
A	30	0.29	1.72	0.50	−3.30	4.2	0.32	1.22	0.71
B	30	−0.22	1.07	−0.20	−2.00	2.5	0.20	0.76	0.72

**Table 3:** The inter-rater agreement for the total cervical translation mobility in the sagittal plane. Mean values, standard deviations (SD), medians, minimum and maximum ranges of motion for rater A and B and for the differences between A and B are presented, as well as the limits of agreement (individual reference interval (mean ± 1.96 ∗ SD)), the *P* values for the systematic differences, and the intraclass correlation coefficients (ICC).

Measurement nr	*N*	Rater A					Rater B					Difference between rater A and B								ICC
					Range					Range					Range		95% Limits of agreement		Systematic difference
		Mean	SD	Median	Min	Max	Mean	SD	Median	Min	Max	Mean	SD	Median	Min	Max	Lower	Higher	*P* value
1	30	9.5	2.5	9.7	5.4	14.0	8.8	1.2	9.0	6.2	11.0	0.8	1.9	1.0	−3.6	3.8	−2.9	4.5	0.036	0.53
2	30	9.2	2.1	9.3	4.4	13.2	9.0	1.7	8.8	6.0	12.0	0.3	1.5	0.1	−3.0	3.8	−2.7	3.3	0.412	0.67
